# Sexual orientation and gender identity: review of concepts, controversies and their relation to psychopathology classification systems

**DOI:** 10.3389/fpsyg.2015.01511

**Published:** 2015-10-01

**Authors:** Carla Moleiro, Nuno Pinto

**Affiliations:** Instituto Universitário de Lisboa ISCTE-IULCIS, Lisboa, Portugal

**Keywords:** sexual orientation, gender identity, transgender, discrimination, psychopathology, mental health care

## Abstract

Numerous controversies and debates have taken place throughout the history of psychopathology (and its main classification systems) with regards to sexual orientation and gender identity. These are still reflected on present reformulations of gender dysphoria in both the Diagnostic and Statistical Manual and the International Classification of Diseases, and in more or less subtle micro-aggressions experienced by lesbian, gay, bisexual and *trans* patients in mental health care. The present paper critically reviews this history and current controversies. It reveals that this deeply complex field contributes (i) to the reflection on the very concept of mental illness; (ii) to the focus on subjective distress and person-centered experience of psychopathology; and (iii) to the recognition of stigma and discrimination as significant intervening variables. Finally, it argues that sexual orientation and gender identity have been viewed, in the history of the field of psychopathology, between two poles: gender transgression and gender variance/fluidity.

## Concepts and Definitions

Concepts and definitions that refer to sexual orientation and gender identity are an evolving field. Many of the terms used in the past to describe LGBT people, namely in the mental health field, are now considered to be outdated and even offensive.

Sexual orientation refers to the sex of those to whom one is sexually and romantically attracted (American Psychological Association, 2012). Nowadays, the terms ‘lesbian’ and ‘gay’ are used to refer to people who experience attraction to members of the same sex, and the term ‘bisexual’ describe people who experience attraction to members of both sexes. It should be noted that, although these categories continue to be widely used, sexual orientation does not always appear in such definable categories and, instead, occurs on a continuum (American Psychological Association, 2012), and people perceived or described by others as LGB may identify in various ways (D’Augelli, 1994).

The expression gender identity was coined in the middle 1960s, describing one’s persistent inner sense of belonging to either the male and female gender category (Money, 1994). The concept of gender identity evolved over time to include those people who do not identify either as female or male: a “person’s self concept of their gender (regardless of their biological sex) is called their gender identity” (Lev, 2004, p. 397). The American Psychological Association (2009a, p. 28) described it as: “the person’s basic sense of being male, female, or of indeterminate sex.” For decades, the term ‘transsexual’ was restricted for individuals who had undergone medical procedures, including genital reassignment surgeries. However, nowadays, ‘transsexual’ refers to anyone who has a gender identity that is incongruent with the sex assigned at birth and therefore is currently, or is working toward, living as a member of the sex other than the one they were assigned at birth, regardless of what medical procedures they may have undergone or may desire in the future (e.g., Serano, 2007; American Psychological Association, 2009a; Coleman et al., 2012). In this paper we use the prefix *trans* when referring to transsexual people.

Since the 1990’s the word transgender has been used primarily as an umbrella term to describe those people who defy societal expectations and assumptions regarding gender (e.g., Lev, 2004; American Psychological Association, 2009a). It includes people who are transsexual and intersex, but also those who identify outside the female/male binary and those whose gender expression and behavior differs from social expectations. As in the case of sexual orientation, people perceived or described by others as transgender – including transsexual men and women – may identify in various ways (e.g., Pinto and Moleiro, 2015).

## Discrimination and Impact on Mental Health

Lesbian, gay, bisexual and transgender people often suffer from various forms of discrimination, stigma and social exclusion – including physical and psychological abuse, bullying, persecution, or economic alienation (United Nations, 2011; Bostwick et al., 2014; European Union Agency for Fundamental Rights, 2014). Moreover, experiences of discrimination may occur in various areas, such as employment, education and health care, but also in the context of meaningful interpersonal relationships, including family (e.g., Milburn et al., 2006; Feinstein et al., 2014; António and Moleiro, 2015). Accordingly, several studies strongly suggest that experiences of discrimination and stigmatization place LGBT people at higher risk for mental distress (Cochran and Mays, 2000; Dean et al., 2000; Cochran et al., 2003; Meyer, 2003; Shilo, 2014).

For example, LGB populations may be at increased risk for suicide (Hershberger and D’Augelli, 1995; Mustanski and Liu, 2013), traumatic stress reactions (D’Augelli et al., 2002), major depression disorders (Cochran and Mays, 2000), generalized anxiety disorders (Bostwick et al., 2010), or substance abuse (King et al., 2008). In addition, transgender people have been identified as being at a greater risk for developing: anxiety disorders (Hepp et al., 2005; Mustanski et al., 2010); depression (Nuttbrock et al., 2010; Nemoto et al., 2011); social phobia and adjustment disorders (Gómez-Gil et al., 2009); substance abuse (Lawrence, 2008); or eating disorders (Vocks et al., 2009). At the same time, data on suicide ideation and attempts among this population are alarming: Maguen and Shipherd (2010) found the percentage of attempted suicides to be as high as 40% in transsexual men and 20% in transsexual women. Nuttbrock et al. (2010), using a sample of 500 transgender women, found that around 30% had already attempted suicide, around 35% had planned to do so, and close to half of the participants expressed suicide ideation. In particular, adolescence has been identified as a period of increased risk with regard to the mental health of transgender and transsexual people (Dean et al., 2000).

In sum, research clearly recognizes the role of stigma and discrimination as significant intervening variables in psychopathology among LGBT populations. Nevertheless, the relation between sexual orientation or gender identity and stress may be mediated by several variables, including social and family support, low internalized homophobia, expectations of acceptance vs. rejection, contact with other LGBT people, or religiosity (Meyer, 2003; Shilo and Savaya, 2012; António and Moleiro, 2015; Snapp et al., 2015). Thus, it seems important to focus on subjective distress and in a person-centered experience of psychopathology.

## On the History of Homosexuality and Psychiatric Diagnoses

While nowadays we understand that higher rates of psychological distress among LGB people are related to their minority status and to discrimination, by the early 20th century, psychiatrists mostly regarded homosexuality as pathological *per se*; and in the mid-20th century psychiatrics, physicians, and psychologists were trying to “cure” and change homosexuality (Drescher, 2009). In 1952, the American Psychiatric Association published its first edition of the *Diagnostic and Statistical Manual* (DSM-I), in which homosexuality was considered a “sociopathic personality disturbance.” In DSM-II, published in 1968, homosexuality was reclassified as a “sexual deviation.” However, in December 1973, the American Psychiatric Association’s Board of Trustees voted to remove homosexuality from the DSM.

The most significant catalyst to homosexuality’s declassification as a mental illness was lesbian and gay activism, and its advocacy efforts within the American Psychiatric Association (Drescher, 2009). Nevertheless, during the discussion that led to the diagnostic change, APA’s Nomenclature Committee also wrestled with the question of what constitutes a mental disorder. Concluding that “they [mental disorders] all regularly caused subjective distress or were associated with generalized impairment in social effectiveness of functioning” (Spitzer, 1981, p. 211), the Committee agreed that homosexuality by itself was not one.

However, the diagnostic change did not immediately end the formal pathologization of some presentations of homosexuality. After the removal of the “homosexuality” diagnosis, the DSM-II contained the diagnosis of “sexual orientation disturbance,” which was replaced by “ego dystonic homosexuality” in the DSM-III, by 1980. These diagnoses served the purpose of legitimizing the practice of sexual “conversion” therapies among those individuals with same-sex attractions who were distressed and reported they wished to change their sexual orientation (Spitzer, 1981; Drescher, 2009). Nonetheless, “ego-dystonic homosexuality” was removed from the DSM-III-R in 1987 after several criticisms: as formulated by Drescher (2009, p. 435): “should people of color unhappy about their race be considered mentally ill?”

The removal from the DSM of psychiatric diagnoses related to sexual orientation led to changes in the broader cultural beliefs about homosexuality and culminated in the contemporary civil rights quest for equality (Drescher, 2012). In contrast, it was only in 1992 that the World Health Organization (World Health Organization, 1992) removed “homosexuality” from the International Classification of Diseases (ICD-10), which still contains a diagnosis similar to “ego-dystonic homosexuality.” However, this is expected to change in the next revision, planned for publication in 2017 (Cochran et al., 2014).

## Controversies on Gender Dysphoria and (Trans)Gender Diagnoses

Mental health diagnoses that are specific to transgender and transsexual people have been highly controversial. In this domain, the work of Harry Benjamin was fundamental for *trans* issues internationally, through the Harry Benjamin International Gender Dysphoria Association (presently, the World Professional Association for Transgender Health, WPATH). In the past few years, there has been a vehement discussion among interested professionals, *trans* and LGBT activists, and human rights groups concerning the reform or removal of (trans)gender diagnoses from the main health diagnostic tools. However, discourses on this topic have been inconclusive, filled with mixed messages and polarized opinions (Kamens, 2011). Overall, mental health diagnoses which are specific to transgender people have been criticized in large part because they enhance the stigma in a population which is already particularly stigmatized (Drescher, 2013). In fact, it has been suggested that the label “mental disorder” is the main factor underlying prejudice toward *trans* people (Winter et al., 2009).

The discussion reached a high point during the recent revision process of the DSM-5 (American Psychiatric Association, 2013), in which the diagnosis of “gender identity disorder” was revised into one of “gender dysphoria.” Psychiatric diagnosis was thus limited to those who are, in a certain moment of their lives, distressed about living with a gender assignment they experience as incongruent with their gender identity (Drescher, 2013). The change of criteria and nomenclature “is less pathologizing as it no longer implies that one’s identity is disordered” (DeCuypere et al., 2010, p. 119). In fact, gender dysphoria is not a synonym for transsexuality, nor should it be used to describe transgender people in general (Lev, 2004); rather, “[it] is a clinical term used to describe the symptoms of excessive pain, agitation, restless, and malaise that gender-variant people seeking therapy often express” (Lev, 2004, p. 910). Although the changes were welcomed (e.g., DeCuypere et al., 2010; Lev, 2013), there are still voices arguing for the “ultimate removal” (Lev, 2013, p. 295) of gender dysphoria from the DSM. Nevertheless, attention is presently turned to the ongoing revision of the ICD. Various proposals concerning the revision of (trans)gender diagnoses within ICD have been made, both originating from transgender and human rights groups (e.g., Global Action for Trans^∗^ Equality, 2011; TGEU, 2013) and the health profession community (e.g., Drescher et al., 2012; World Professional Association for Transgender Health, 2013). These include two main changes: the reform of the diagnosis of transsexualism into one of “gender incongruence”; and the change of the diagnosis into a separate chapter from the one on “mental and behavioral disorders.”

## Mental Health Care Reflecting Controversies

There is evidence that LGBT persons resort to psychotherapy at higher rates than the non-LGBT population (Bieschke et al., 2000; King et al., 2007); hence, they may be exposed to higher risk for harmful or ineffective therapies, not only as a vulnerable group, but also as frequent users.

Recently, there has been a greater concern in the mental health field oriented to the promotion of the well-being among non-heterosexual and transgender people, which has paralleled the diagnostic changes. This is established, for instance, by the amount of literature on gay and lesbian affirmative psychotherapy which has been developed in recent decades (e.g., Davis, 1997) and, also, by the fact that major international accrediting bodies in counseling and psychotherapy have identified the need for clinicians to be able to work effectively with minority clients, namely LGBT people. The APA’s guidelines for psychotherapy with lesbian, gay, and bisexual client (American Psychological Association, 2000, 2012) are a main reference. These ethical guidelines highlight, among several issues, the need for clinicians to recognize that their own attitudes and knowledge about the experiences of sexual minorities are relevant to the therapeutic process with these clients and that, therefore, mental health care providers must look for appropriate literature, training, and supervision.

However, empirical research also reveals that some therapists still pursue less appropriate clinical practices with LGBT clients. In a review of empirical research on the provision of counseling and psychotherapy to LGB clients, Bieschke et al. (2006) encountered an unexpected recent explosion of literature focused on “conversion therapy.” There are, in fact, some mental health professionals that still attempt to help lesbian, gay, and bisexual clients to *become* heterosexual (Bartlett et al., 2009), despite the fact that a recent systematic review of the peer-reviewed journal literature on sexual orientation change efforts concluded that “efforts to change sexual orientation are unlikely to be successful and involve some risk of harm” (American Psychological Association, 2009b, p. 1).

Moreover, there is evidence of other forms of inappropriate (while less blatant) clinical practices with LGBT clients (e.g., Garnets et al., 1991; Jordan and Deluty, 1995; Liddle, 1996; Hayes and Erkis, 2000). Even those clinicians who intend to be affirmative and supportive of LGBT individuals can reveal subtle heterosexist bias in the work with these clients (Pachankis and Goldfried, 2004). Examples of such micro-aggressions (Sue, 2010) might be automatically assuming that a client is heterosexual, trying to explain the etiology of the client’s homosexuality, or focusing on the sexual orientation of a LGB client despite the fact that this is not an issue at hand (e.g., Shelton and Delgado-Romero, 2011). Heterosexual bias in counseling and psychotherapy may manifest itself also in what Brown (2006, p. 350) calls “sexual orientation blindness,” i.e., struggling for a supposed neutrality and dismissing the specificities related to the minority condition of non-heterosexual clients. This conceptualization of the human experience mostly in heterosexual terms, found in the therapeutic setting, does not seem to be independent of psychotherapist’s basic training and the historical heterosexist in the teaching of medicine and psychology (Simoni, 1996; Alderson, 2004).

With regards to the intervention with *trans* people, for decades the mental health professionals’ job was to sort out the “true” transsexuals from all other transgender people. The former would have access to physical transition, and the later would be denied any medical intervention other than psychotherapy. By doing this, whether deliberately or not, professionals – acting as gatekeepers – pursued to ‘ensure that most people who did transition would not be “gender-ambiguous” in any way’ (Serano, 2007, p. 120). Research shows that currently *trans* people still face serious challenges in accessing health care, including those related to inappropriate gatekeeping (Bockting et al., 2004; Bauer et al., 2009). Some mental health professionals still focus on the assessment of attributes related to identity and gender expressions, rather than on the distress with which *trans* people may struggle with (Lev, 2004; Serano, 2007). Hence, *trans* people may feel the need to express a personal narrative consistent with what they believe the clinicians’ expectations to be, for accessing hormonal or surgical treatments (Pinto and Moleiro, 2015). Thus, despite the revisions of (trans)gender diagnoses within the DSM, more recent diagnoses seem to still be used as if they were identical with the diagnosis of transsexualism – in a search for the “true transsexual” (Cohen-Kettenis and Pfäﬄin, 2010). It seems clear that social and cultural biases have significantly influenced – and still do – diagnostic criteria and the access to hormonal and surgical treatments for *trans* people.

## Conclusion

Controversies and debates with regards to medical classification of sexual orientation and gender identity contribute to the reflection on the very concept of mental illness. The agreement that mental disorders cause subjective distress or are associated with impairment in social functioning was essential for the removal of “homosexuality” from the DSM in the 1970s (Spitzer, 1981). Moreover, (trans)gender diagnoses constitute a significant dividing line both within *trans* related activism (e.g., Vance et al., 2010) and the health professionals’ communities (e.g., Ehrbar, 2010). The discussion has taken place between two apposite positions: (1) trans(gender) diagnoses should be removed from health classifying systems, because they promote the pathologization and stigmatization of gender diversity and enhance the medical control of *trans* people’s identities and lives; and (2) trans(gender) diagnoses should be retained in order to ensure access to care, since health care systems rely on diagnoses to justify medical treatment – which many *trans* people need. In fact, *trans* people often describe experiences of severe distress and argue for the need for treatments and access to medical care (Pinto and Moleiro, 2015), but at the same time reject the label of mental illness for themselves (Global Action for Trans^∗^ Equality, 2011; TGEU, 2013). Thus, it may be important to understand how the debate around (trans)diagnoses may be driven also by a history of undue gatekeeping and by stigma involving mental illness.

The present paper argues that sexual orientation and gender identity have been viewed, in the history of the field of psychopathology, between two poles: gender transgression and gender variance/fluidity.

On the one hand, aligned with a position of “transgression” and/or “deviation from a norm,” people who today are described as LGBT were labeled as mentally ill. Inevitably, classification systems reflect(ed) the existing social attitudes and prejudices, as well as the historical and cultural contexts in which they were developed (Drescher, 2012; Kirschner, 2013). In that, they often failed to differentiate between mental illness and socially non-conforming behavior or fluidity of gender expressions. This position and the historical roots of this discourse are still reflected in the practices of some clinicians, ranging from “conversion” therapies to micro-aggressions in the daily lives of LGBT people, including those experienced in the care by mental health professionals.

On the other hand, lined up with a position of gender variance and fluidity, changes in the diagnostic systems in the last few decades reflect a broader respect and value of the diversity of human sexuality and of gender expressions. This position recognizes that the discourse and practices coming from the (mental) health field may lead to changes in the broader cultural beliefs (Drescher, 2012). As such, it also recognizes the power of medical classifications, health discourses and clinical practices in translating the responsibility of fighting discrimination and promoting LGBT people’s well-being.

In conclusion, it seems crucial to emphasize the role of specific training and supervision in the development of clinical competence in the work with sexual minorities. Several authors (e.g., Pachankis and Goldfried, 2004) have argued for the importance of continuous education and training of practitioners in individual and cultural diversity competences, across professional development. This is in line with APA’s ethical guidelines (American Psychological Association, 2000, 2012), and it is even more relevant when we acknowledge the significant and recent changes in this field. Furthermore, it is founded on the very notion that LGBT competence assumes clinicians ought to be aware of their own personal values, attitudes and beliefs regarding human sexuality and gender diversity in order to provide appropriate care. These ethical concerns, however, have not been translated into training programs in medicine and psychology in a systematic manner in most European countries, and to the mainstreaming of LGBT issues (Goldfried, 2001) in psychopathology.

## Conflict of Interest Statement

The authors declare that the research was conducted in the absence of any commercial or financial relationships that could be construed as a potential conflict of interest.
